# Modulation of the Pol II CTD Phosphorylation Code by Rac1 and Cdc42 Small GTPases in Cultured Human Cancer Cells and Its Implication for Developing a Synthetic-Lethal Cancer Therapy

**DOI:** 10.3390/cells9030621

**Published:** 2020-03-04

**Authors:** Bo Zhang, Xuelin Zhong, Moira Sauane, Yihong Zhao, Zhi-Liang Zheng

**Affiliations:** 1 Department of Biological Sciences, Lehman College, City University of New York, Bronx, NY 10468, USA; 2 Department of Biology, Wenzhou-Kean University, Wenzhou, Zhejiang 325060, China; 3 Biology PhD Program, Graduate School and University Center, City University of New York, New York, NY 10016, USA; 4 Center of Alcohol and Substance Use Studies and Department of Applied Psychology, Graduate School of Applied and Professional Psychology, Rutgers University, Piscataway, NJ 08854, USA

**Keywords:** Rho, Rac1, Cdc42, Pol II, CTD code, CDK7, DOCK4, DOCK9, THZ1, Torin1

## Abstract

Rho GTPases, including Rho, Cdc42, Rac and ROP subfamilies, are key signaling molecules in RNA polymerase II (Pol II) transcriptional control. Our prior work has shown that plant ROP and yeast Cdc42 GTPases similarly modulate Ser2 and Ser5 phosphorylation status of the C-terminal domain (CTD) of the Pol II largest subunit by regulating CTD phosphatase degradation. Here, we present genetic and pharmacological evidence showing that Cdc42 and Rac1 GTPase signaling modulates a similar CTD Ser2 and Ser5 phosphorylation code in cultured human cancer cells. While siRNA knockdown of *Cdc42* and *Rac1*, respectively, in HeLa cells increased the level of CTD Ser phosphatases RPAP2 and FCP1, they both decreased the level of CTD kinases CDK7 and CDK13. In addition, the protein degradation inhibitor MG132 reversed the effect of THZ1, a CDK7 inhibitor which could decrease the cell number and amount of CDK7 and CDK13, accompanied by a reduction in the level of CTD Ser2 and Ser5 phosphorylation and DOCK4 and DOCK9 (the activators for Rac1 and Cdc42, respectively). Conversely, treatments of Torin1 or serum deprivation, both of which promote protein degradation, could enhance the effect of THZ1, indicating the involvement of protein degradation in controlling CDK7 and CDK13. Our results support an evolutionarily conserved signaling shortcut model linking Rho GTPases to Pol II transcription across three kingdoms, Fungi, Plantae and Animalia, and could lead to the development of a potential synthetic-lethal strategy in controlling cancer cell proliferation or death.

## 1. Introduction

Regulation of gene expression is essential for cellular activities in response to intracellular cues and extracellular signals. Ras superfamily small GTPases, including Ras and Rho families, act as key signaling switches in regulating gene expression, and consequently deregulation of these signaling GTPases is frequently associated with many diseases including cancer [1,2,3,4,5]. There are two models regarding Ras and Rho GTPase control of RNA polymerase (Pol) II transcription: the well-known classical or “indirect” model, and the recently re-emerged direct or “shortcut” model [6]. The classical model has been described in genetics and cell biology textbooks and literature and presented as a paradigm in oncogenic signaling networks. In this model, activation of Ras or Rho GTPases causes a cascade of MAP kinase phosphorylation events in the cytoplasm, leading to translocation of MAPK into the nucleus and activation of sequence-specific transcription factors. In contrast, the shortcut model allows activated GTPases to target protein kinase A or proteasome to regulate a component of Mediator [7] or Pol II [8]. Given that the shortcut model does not involve the MAP kinase cascade and instead directly regulates Pol II or its components, this model has a potential of efficiently regulating transcription so as to rapidly bring about the broad changes in gene expression in response to dynamic intracellular and extracellular changes [6,7,8].

In our prior work, we have found that Rho family GTPases, including ROP2/4 in the *Arabidopsis* model plants and Cdc42 in fission yeast, modulate the phosphorylation status of Ser2 and Ser5 in the C-terminal domain (CTD) of RPB1, the largest subunit of Pol II, effecting gene expression and the control of cell shape, cell size and cell number [8]. The Pol II CTD contains various number of the heptad peptide (Y^1^S^2^P^3^T^4^S^5^P^6^S^7^) repeat, ranging from 29 repeats in fission yeast and 34 in *Arabidopsis* to 52 in humans [9,10,11,12]. Dynamic post-translational modifications of these seven residues in each repeat, in particular phosphorylation of Ser2 and Ser5, constitutes a very complex pattern called the “CTD code”, which is critical for completing key steps of the transcription cycle [9,10,11,12]. The CTD code is created and maintained by several CTD kinases and CTD phosphatases. Using genetic and biochemical approaches, we have shown that elevation of the CTD Ser2 and Ser5 phosphorylation status caused by activation of ROP2 and Cdc42 GTPases is mediated by degradation of CTD phosphatases, CPL1 in *Arabidopsis* or Fcp1 in yeast [8]. However, whether these Rho GTPases also regulate CTD kinases remains to be investigated.

The similarity in the CTD Ser2 and Ser5 phosphorylation pattern and its underlying biochemical mechanism (degradation of CTD phosphatases) observed in both *Arabidopsis* and yeast strongly indicate that the Rho-Pol II shortcut model of transcriptional control is evolutionarily conserved in eukaryotic organisms. Therefore, we hypothesize that human cells likely use Rho family GTPases to modulate a similar CTD code. However, humans, plants and yeast are separated by a billion years of evolution, and thus it is also possible that human cells have adopted an overlapping and yet somehow distinct mechanism in the Rho signaling-mediated CTD code modulation. To test these hypotheses, we used various GTPase inhibitors and knockdown of *Rac1* and *Cdc42* to study the Pol II CTD Ser2 and Ser5 phosphorylation patterns. Our results suggest that Rac1 and Cdc42 GTPase signaling in cultured human cancer cells similarly modulates the CTD Ser2 and Ser5 phosphorylation status. In addition, while these two GTPases suppress different CTD phosphatases, they both increase the level of CTD kinases CDK7 and CDK13. Interestingly, our results from the combined treatments of a covalent CDK7 inhibitor THZ1 and chemicals that inhibit or stimulate protein degradation imply a potential role for THZ1 in degrading CDK7, CDK13 and activators of Rac1 (DOCK4) and Cdc42 (DOCK9), which can potentially lead to lower activity of Rac1 and Cdc42 and thus forms a possible feedback regulatory loop in the proposed Rho-Pol II signaling shortcut model.

## 2. Materials and Methods

### 2.1. Human Cancer Cell Cultures

Human prostate DU145 and cervical carcinoma HeLa cell lines were acquired from the American Type Culture Collection (ATCC), maintained per ATCC protocols and utilized within six months of thawing. Cell lines were grown in a humidified atmosphere at 37 °C with 5% CO_2_, using MEM medium (Gibco) supplemented with 10% fetal bovine serum (Corning) and 1% Pen Strep antibiotics (Gibco). The culture medium was replaced every other day.

### 2.2. siRNA Transfection

*Cdc42* and *Rac1* siRNA were designed and supplied by OriGene Technologies. For *Cdc42* silencing, a mixture of 3 unique 27-mer (rArCrArArArUrUrUrCrCrArUrCrGrGrArArUrArUrGrUrACC, rCrCrArCrArArArCrArGrArUrGrUrArUrUrUrCrUrArGrUCT and rGrGrArGrArArCrCrArUrArUrArCrUrCrUrUrGrGrArCrUTT) siRNA duplexes (OriGene, SR300714) was used. For *Rac1* silencing, a mixture of 3 unique 27-mer (rGrGrArArCrUrArArArCrUrUrGrArUrCrUrUrArGrGrGrATG, rArCrArUrUrGrUrArCrUrGrUrArArUrGrGrArGrUrGrArGCG and rGrUrArGrUrUrCrUrCrArGrArUrGrCrGrUrArArArGrCrAGA) siRNA duplexes (OriGene, SR303958) was used. The SiLentFect (BIO-RAD) lipid reagent was used for transfection, and the experiments were performed by following the recommended protocol.

### 2.3. Inhibitor Treatments

All inhibitors were dissolved in DMSO, and the control contained the DMSO only. Ten µM farnesylthiosalicylic acid (FTS; Sigma, SML1166), 5 µM Y16 (Sigma,SML0873), 10 µM Ehop-016 (Sigma, SML0526) and 2 µM ML141 (Sigma, SML0407), were used as Ras, Rho, Rac and Cdc42 GTPase inhibitors, respectively, for 48 hr in HeLa and DU145 cells. For THZ1 and MG132 treatments, HeLa cells were treated by 100 nM THZ1 (MCE, 80013) and 40 µM MG132 (ChemCruz, sc-201270) for 8 hr. For Torin1 and serum depletion treatments, HeLa cells were treated with 100 nM Torin1 (MCE, HY-13003), serum deprivation, or their combination with 100 nM THZ1 for 8 hr for analyzing protein level using Western blot and for 24 or 48 hr for observing cell number.

### 2.4. Western Blot

Cells were collected and disrupted with RIPA buffer (Pierce IP lysis buffer; Thermo Scientific™; Cat. #87787). Total protein levels were quantified using Pierce™ BCA Protein Assay Kit (Thermo Scientific™). Standard Western blot procedure was used, i.e., protein samples were separated by SDS-PAGE and electrophoretically transferred onto PVDF membranes. The following primary antibodies were used for protein detection: anti-Ser5p (Abcam, ab5131), anti-Ser2p (Abcam, ab5095), anti-Ser7p (Active Motif, Clone 4E12), anti-CTD (Abcam, ab26721), anti-α-tubulin (Sigma, T6074), anti-DOCK4 (Bethyl Laboratories, A302-263A), anti-DOCK9 (Bethyl Laboratories, A300-530A), anti- CTDP1/FCP1 (Bethyl Laboratories, A301-172A), anti- RPAP2 (Proteintech, 17401-1-AP), anti-Cdc42 (Santa cruz, B-8, sc-8401), anti-RAC1 (Proteintech, 24072-1-AP), anti-CDK7 (Bethyl Laboratories, A300-405A) and anti- CDC2L5/CDK13 (Bethyl Laboratories, A301-458A). The following secondary antibodies were used: stabilized Goat anti-Mouse HRP-Conjugated (Pierce, 1858413), stabilized Goat anti-Rabbit HRP-Conjugated (Pierce, 1858415) and Goat anti-Rat HRP Conjugated (Thermo, 31470). After antibody incubation, the membrane was incubated with the chemiluminescent substrate (Thermo, 34096) by following the manufacturer’s protocol. Signal intensity was acquired by exposure to X-ray film. For membrane stripping and re-probing, a mild stripping method was used by following Abcam’s protocol.

### 2.5. Quantitative Analysis of Western Blots and Cell Counting

For protein quantification, ImageJ software was used to quantify the bands in Western blots. The ratio of each protein band relative to tubulin, an internal loading control, was derived. The relative abundance of proteins in treatment groups was obtained by normalizing the ratio against that in the control group, which was set as 1.0. Two-way analysis of variance (ANOVA) with protein and treatment interaction was performed. Multiple pairwise comparisons between treatment group differences for each protein were obtained using the Tukey Honest Significant Differences method with the R function TukeyHSD(). For cell counting, cell images were captured with a Nikon confocal spinning disk microscope. Each image stands for one separate vision field. For each treatment, six individual images were used for cell counting, without any image cropping or modification. Hela cells with intact morphology and clear structure were counted. The average cell number of all replicates in the control group was set as 100, and the treatment groups were then normalized against the control average. One-way ANOVA analysis was used to assess mean cell number differences between treatment groups and adjusted *p*-values were obtained using the R function TukeyHSD(). All the data are presented as means and standard error of means (SEM), with * indicating adjusted *p*<0.05 and ** adjusted *p*<0.01.

## 3. Results

### 3.1. Treatments by Rho/Rac/Cdc42 Inhibitors Decreased the Pol II CTD Ser2 and Ser5 Phosphorylation Levels

**Figure 1:**
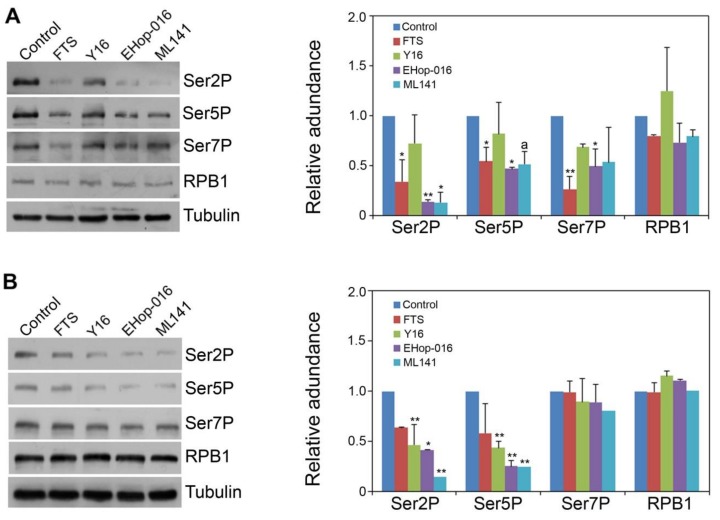
Inhibitors of small GTPases reduced CTD Ser2P and Ser5P levels. Cells were treated with various inhibitors for 48 hr, and the proteins were analyzed by Western blot using Ser5P-, Ser2P- and Ser7P-specific antibodies. (**A**) HeLa cells. (**B**) DU145 cells. FTS, farnesylthiosalicylic acid, a Ras antagonist. Y16, a RhoA inhibitor. Ehop-016, a Rac1 and Rac3 inhibitor. ML141, a Cdc42 inhibitor. Tubulin, the protein loading control. Quantitative analysis of the Western blot results from two independent biological replicates was shown. Protein level in the control was set as 1.0. Significance level is indicated by * (*p* < 0.05) or ** (*p* < 0.01) vs. control. The letter *a* above the column indicates a marginal difference (*p* < 0.10).

To gain initial insights into the possible modulation of the CTD code by Rho signaling in human cells, we used the relatively specific inhibitors for RhoA, Rac and Cdc42 in two human cancer cell lines: the prostate cancer DU145 cell line, and the cervical carcinoma HeLa cell line. We also included Ras GTPase inhibitor in the experiment as a comparison, given that Ras and Rho family GTPases all have been implicated in cancer [2,3,4,5]. As shown in Figure 1, the inhibitors overall had some varying impacts in CTD Ser2 phosphorylation (Ser2P) and Ser5 phosphorylation (Ser5P) levels, depending on the types of inhibitors and cancer cell lines. For example, the Ras antagonist farnesylthiosalicylic acid (FTS) [13] was effective to reduce Ser2P, Ser5P and Ser7P levels in HeLa cells (Figure 1A), but the difference in DU145 was not statistically significant (Figure 1B). In contrast, the RhoA inhibitor Y16 [14] strongly reduced Ser2P and Ser5P levels in DU145 cells but had no significant differences in HeLa cells compared to the control. Interestingly, Ehop-016, a Rac1 and Rac3 inhibitor [15], significantly reduced Ser2P and Ser5P levels in both cancer cell lines, although it also slightly reduced Ser7P in HeLa cells. ML141, a Cdc42 inhibitor [16], was also effective in reducing Ser2P and Ser5P levels in both cancer cell lines, except that the difference in Ser5P between control and ML141 was only marginal in HeLa cells. Overall, while inhibitors of Ras and RhoA showed differential effects in the CTD Ser2 and Ser5 phosphorylation status depending on the cancer cell lines, inhibitors of Rac1 and Cdc42 more consistently reduced Ser2P and Ser5P levels in both cell lines without greatly affecting Ser7P and total RPB1 protein accumulation. Therefore, despite some variations in different cancer cell lines, these inhibitor treatment results strongly indicate that activity of the members from each of Rho subfamilies modulates the CTD Ser2 and Ser5 phosphorylation code in cultured human cancer cells.

### 3.2. Rac1 and Cdc42 Knockdown by siRNA Decreased CTD Ser2P and Ser5P Levels and Differentially Affected Levels of CTD Phosphatases and Kinases

**Figure 2:**
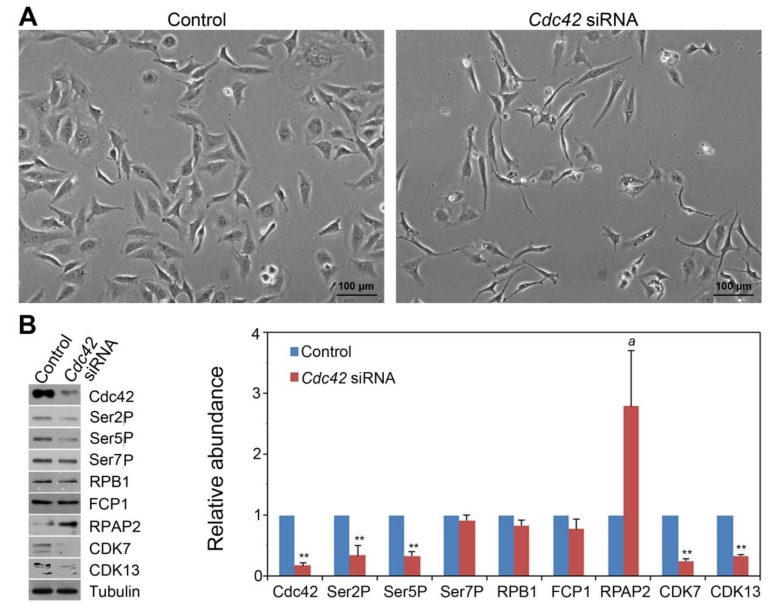
siRNA-based *Cdc42* knockdown modulated the CTD phosphorylation code and affected the level of CTD phosphatases and kinases in HeLa cells. (**A**) HeLa cells 48 hr after *Cdc42* siRNA transfection. (**B**) Western blot showing the levels of Cdc42, Ser2P, Ser5P, Ser7P and CTD phosphatases (FCP1 and RPAP2) and kinases (CDK7 and CDK13) in *Cdc42* siRNA-transfected HeLa cells. Tubulin was used as the protein loading control. Quantitative analysis of the Western blot results from two independent biological replicates was shown. Protein level in the control was set as 1.0. Significance level is indicated by * (*p* < 0.05) or ** (*p* < 0.01) vs. control. The letter *a* above the column indicates a marginal difference (*p* = 0.058) compared to control.

To substantiate the finding regarding the role of Rac1 and Cdc42 signaling in the CTD phosphorylation code modulation, we carried out the siRNA transfection-based knockdown studies for *Cdc42* and *Rac1*, respectively. As shown in Figure 2A, *Cdc42* siRNA treatment altered the shape of HeLa cells, leading to the formation of some thinner, elongated cells. Such cell shape phenotype has been reported in *Cdc42*-deficient fibroblastoid cells by others [17]. There was no statistical difference in cell number between control and siRNA treatment (Supplementary Figure S1A). Western blot analysis showed that *Cdc42* siRNA reduced Cdc42 protein level by 80% (Figure 2B), indicating the effectiveness of siRNA-mediated knockdown. We found that siRNA treatment decreased Ser2P and Ser5P levels by approximately 70%, without affecting Ser7P and total RPB1. This result was consistent with the Cdc42-specific inhibitor experiment (Figure 1A).

To determine whether the decrease of the Ser2P and Ser5P level is related to the alteration of CTD phosphatases or CTD kinases, we assessed the levels of two CTD phosphatases (FCP1 and RPAP2) and two CTD kinases (CDK7 and CDK13). We found that the protein level for FCP1, a Ser2 and Ser5 phosphatase [18], which is orthologous to yeast Fcp1, did not increase in *Cdc42* siRNA-treated cells (Figure 2B). Instead, another CTD phosphatase, RPAP2, which acts at Ser5 [19], had a marginal difference between siRNA-transfected cells and the control (1.9- and 3.7-fold increase in two independent replicates, respectively). In addition, two CTD kinases, CDK7 (which phosphorylates Ser5 and Ser7 [20]) and CDK13 (acting at Ser2 and Ser5 [21]), were both reduced by 3- to 4-fold in *Cdc42* siRNA treated cells. This result indicates that Cdc42 signaling in human cells similarly modulates the CTD Ser2 and Ser5 phosphorylation code as in plant and yeast systems, and yet it does so possibly by inhibiting RPAP2 and activating CDK7 and CDK13.

**Figure 3:**
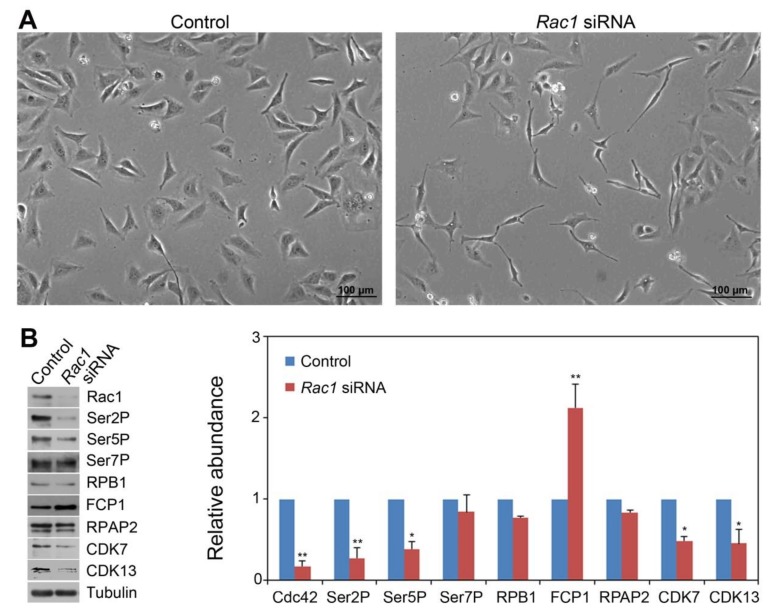
siRNA-based *Rac1* knockdown modulated the CTD phosphorylation code and affected the level of CTD phosphatases and kinases in Hela cells. (**A**) HeLa cells 48 hr after *Rac1* siRNA transfection. (**B**) Western blot showing the levels of Cdc42, Ser2P, Ser5P, Ser7P, FCP1, RPAP2, CDK7 and CDK13 in *Rac1* siRNA-transfected HeLa cells. Tubulin, the protein loading control. Quantitative analysis of the Western blot results from two independent biological replicates was shown. Protein level in the control was set as 1.0. Significance level is indicated by * (*p* < 0.05) or ** (*p* < 0.01) vs. control.

For Rac1, we found that *Rac1* siRNA transfection had a similar effect in the formation of some thin, elongated cells siRNA (Figure 3A) as the *Cdc42* siRNA treatment (Figure 2A), without affecting cell number (Supplementary Figure S1B). Rac1 protein level was reduced by 83% (Figure 3B), demonstrating that siRNA was effective in knocking down *Rac1* expression. Similar to *Cdc42* siRNA, *Rac1* siRNA also reduced Ser2P and Ser5P levels by approximately 60-70% without affecting Ser7P and total RPB1 levels. Therefore, both Cdc42 and Rac1 GTPases are involved in a similar CTD phosphorylation code modulation. However, in contrast to *Cdc42*, *Rac1* siRNA caused a 2.1-fold increase in Fcp1 level without affecting RPAP2 level (Figure 3B). With regard to CDK7 and CDK13, *Rac1* siRNA similarly reduced their levels as *Cdc42* siRNA.

Taken together, the results from our siRNA transfection experiments showed that members in the two subfamilies of Rho family GTPases, Rac1 and Cdc42, modulate the CTD Ser2 and Ser5 phosphorylation status. Furthermore, it is possibly that they act by negatively regulating the accumulation of specific CTD phosphatases and positively regulating the accumulation of CTD kinases CDK7 and CDK13.

### 3.3. Effects of THZ1 in CTD Code Modulation and Accumulation of CDK and DOCK Proteins Can Be Reversed by MG132 Treatment

Since our prior findings have demonstrated that yeast Cdc42 and *Arabidopsis* ROP2 GTPases are involved in proteasome-mediated degradation of CTD phosphatases [8], we tested whether MG132, a proteasome inhibitor, would block the *Cdc42* and *Rac1* siRNA effects. However, we repeatedly observed that mixtures of MG132 and siRNA quickly and severely arrested cell growth, which did not allow us to collect enough cells for Western blot analysis. Thus, we decided to use a highly specific CDK7 inhibitor, THZ1, in combination with MG132. The use of THZ1 was motivated by other groups’ prior studies showing that THZ1 can strongly suppress cancer cell growth by inhibiting Pol II CTD phosphorylation and consequently controlling transcription [22,23,24,25].

**Figure 4:**
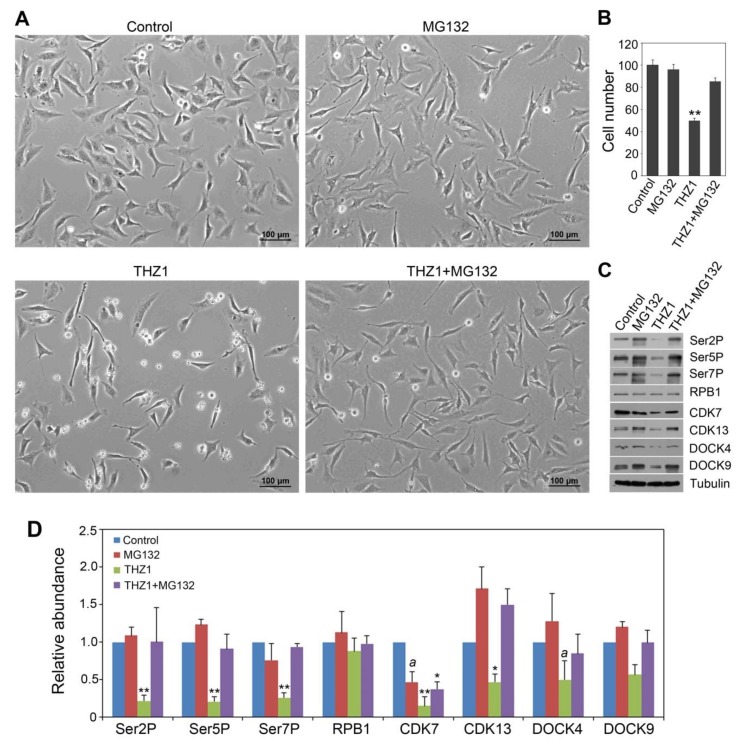
Suppression of the THZ1 effects by MG132 treatment in HeLa cells. (**A**) Cells after 8 hr-treatments of 100 nM THZ1 and 40 µM MG132. (**B**) Quantitative analysis of cell number after treatments. Cell number for the control average is set as 100, and all other treatment groups are normalized to the control. Values are means and SEM of six replicates, with ** indicating significant difference between THZ1 and three other groups (*p* < 0.01). (**C**) Western blot showing the effect of THZ1 suppressed by MG132. Tubulin, the protein loading control. (**D**) Quantitative analysis of the Western blot results from two independent biological replicates. Protein level in the control was set as 1.0. Significance level is indicated by * (*p* < 0.05) or ** (*p* < 0.01) vs. control. The letter *a* above the column indicates a marginal difference (*p* < 0.10) compared to control.

We first assessed whether THZ1 had a similar effect in modulating the CTD code and affecting the CDK targets as *Rac1* and *Cdc42* siRNA knockdown. We observed that 100 nM THZ1 treatment for 8 hr caused HeLa cell growth to be inhibited, resulting in only 50% of the viable cells compared to the no-THZ1 control, with dead cells floating on the surface of the medium (Figure 4A,B). Western blot analysis showed that THZ1 caused approximately 80% reduction in Ser2P and Ser5P levels, without affecting total RPB1 protein accumulation (Figure 4C,D), similar to *Cdc42* and *Rac1* siRNA transfections. However, Ser7P level was also reduced by THZ1, unlike *Rac1* and *Cdc42* siRNA, which did not impact Ser7P level. Surprisingly, THZ1 also reduced the levels of CDK7 (by 85%) and CDK13 (by 53%), a phenomenon not reported by prior studies [22,23,24,25,26] but similar to the effect of *Cdc42* and *Rac1* siRNA-transfection (Figure 2B and Figure 3B). We then studied whether MG132 could oppose the effect of THZ1 in CTD code modulation and CDK7/13 accumulation. Although MG132 alone did not affect Ser2P, Ser5P, Ser7P and total RPB1 levels, there was a trend that combining MG132 and THZ1 (THZ1 + MG132) could increase the levels of Ser2P, Ser5P and Ser7P, compared to THZ1 alone (Figure 4C,D). The lack of statistical differences between MG132 and THZ1 + MG132 indicates that the inhibitory effect of THZ1 could be nullified by MG132. Similarly, although MG132 marginally reduced the CDK7 level and did not affect CDK13, THZ1 + MG132 tended to have higher CDK7 and CDK13 levels, compared to THZ1 alone. Given the lack of differences in CDK7 and CDK13 levels between MG132 and THZ1 + MG132, there remains a possibility that THZ1, an inhibitor of CDK7, might also act by promoting degradation of CDK7 and CDK13, which could be opposed by MG132. Thus, these results consistently suggest an opposing effect of MG132 on THZ1 in controlling the levels of both CTD Ser phosphorylation and CDK7 and CDK13 proteins. Accordingly, we found that THZ1 + MG132 led to more viable cells that remained than THZ1 alone, although MG132 itself under this condition did not affect the HeLa cell number (Figure 4A,B).

In addition, we investigated whether THZ1 also impacted the level of guanine nucleotide exchange factors (GEFs) for Rac1 and Cdc42. This was motivated by our observation that the loss-of-function mutation in *CAE2*, which encodes an *Arabidopsis* plant CTD Ser5 phosphatase called CPL1 [8], increased the level of GTP-bound, active form of ROP GTPase by 4-fold (Supplementary Figure S2). As we have shown previously that the loss of the *CPL1* function results in an increase of the CTD Ser2P and Ser5P level [8], the current result indicates a feedback regulation of ROP2 GTPase activation by the Pol II CTD Ser phosphorylation level. We therefore tested whether inhibition of CTD Ser phosphorylation in HeLa cells by THZ1 would decrease the level of GEFs for Rac1 and Cdc42, and if so, whether this effect could be reversed by MG132. As a first step, we chose to study DOCK4 and DOCK9 among a large family of GEFs, given that DOCK4 and DOCK9 have been demonstrated to respectively activate Rac1 and Cdc42 [27,28,29,30] and that mutations in *DOCK4* [31,32,33,34,35] and *DOCK9/Zizimin1* [36] have been found in a number of cancers. Knockdown of *DOCK4* and *DOCK9* was also reported to reduce cancer growth [37]. As shown in Figure 4C,D, THZ1 caused a 50% reduction in DOCK4 level (a statistically marginal difference) compared to control. For DOCK9, it appeared to have a similar trend of inhibition by THZ1 (although the 30% and 56% reduction in two independent replicates respectively was not statistically significant). As observed for the CTD phosphorylation pattern described above, the combined treatment of MG132 and THZ1 appeared to increase DOCK4 and DOCK9 levels compared to THZ1 alone. In addition, the DOCK4 and DOCK9 levels were similar between MG132 alone and MG132 + THZ1, indicating that MG132 could reverse the THZ1-caused suppression.

### 3.4. Serum Deprivation and Torin1 Treatments Enhanced the THZ1 Effect

**Figure 5:**
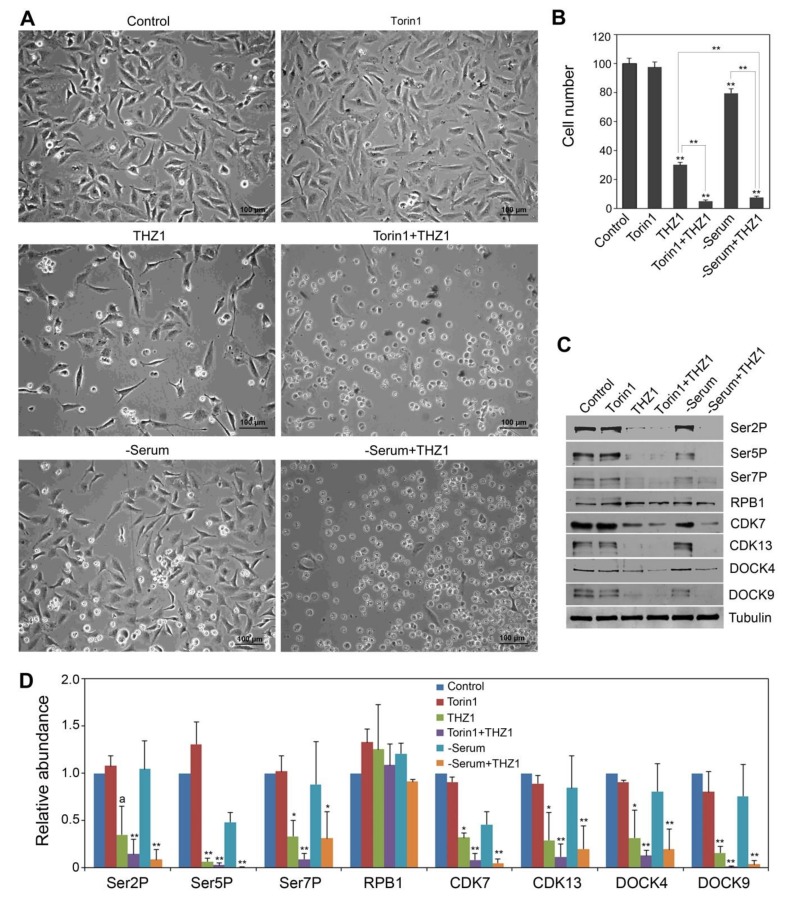
Enhancement of the THZ1 effect by Torin1 and serum deprivation treatments. (**A**) Cells treated with 100 nM THZ1 and 100 nM Torin1 or serum depletion (-Serum) for 24 hr. (**B**) Quantitative analysis of cell number after 24 hr treatments. Cell number for the control average is set as 100, and all other treatment groups are normalized to the control. Values are means and SEM of six replicates, with ** (*p*<0.01) indicating significant differences vs. control, or between treatments as indicated. (**C**) Western blot showing the effect of THZ1 enhanced by Torin1 and serum depletion (-Serum) after 8-hr treatments. Tubulin, the protein loading control. (**D**) Quantitative analysis of the Western blot results from two independent biological replicates. Protein level in the control was set as 1.0. Significance level is indicated by * (*p* < 0.05) or ** (*p* < 0.01) vs. control. The letter *a* above the column indicates a marginal difference (*p* < 0.10) compared to control.

The above observation that proteasome degradation inhibitor MG132 suppresses the effect of THZ1 led us to test whether Torin1 and serum depletion, which have been shown to promote protein degradation [38], could further enhance the impact of THZ1. Our results showed that after 24 hr of treatment, 30% of HeLa cells remained viable with 100 nM THZ1 alone, and the combination of 100 mM Torin1 and 100 nM THZ1 caused most of the cells dead (floating on the surface of the medium) with only 5% of HeLa cells remaining viable (Figure 5A,B). Thus, although Torin1 by itself under this condition did not reduce cell number compared to the control (Figure 5A,B), when combined with THZ1, it resulted in a 6-fold further reduction of cell number compared to THZ1 alone. Serum deprivation in combination with THZ1 had a similar effect as the combined treatment of Torin1 and THZ1 in further reducing the number of viable cells compared with THZ1 alone. Different from Torin1, serum deprivation alone slightly reduced the number of viable cells, which is consistent with the notion that the serum deprivation treatment has a stronger effect in protein degradation than Torin1 [38]. To determine whether lower doses of Torin1 could also exert an enhancement effect on THZ1, we compared the cell number in response to various doses of Torin1 (5, 10, 25 and 50 nM) in combination with 100 nM THZ1 (Supplementary Figure S3). Our linear regression analysis showed that increasing the Torin1 dose was significantly related to decreasing the cell number (adjusted R^2^ = 0.61, *p* < 0.01). Torin1 at low doses (5 and 10 nM) reduced the cell number by approximately 20%–30% compared to the baseline control (0 nM Torin1).

We then used Western blot to study the CTD phosphorylation status and the levels of CDK7, CDK13, DOCK4 and DOCK9. Since the 24 hr treatments of 100 nM Torin1 or serum deprivation in combination with 100 nM THZ1 caused severe cell death, 8-hr-treated cells were instead used in this study. Similar to its lack of effect in cell number, Torin1 alone did not cause a decrease in Ser2P, Ser5P and Ser7P levels (Figure 5C). Although serum deprivation by itself slightly reduced cell number after 24 hr treatment (Figure 5B), it did not affect the levels of Ser2P, Ser5P and Ser7P after 8 hr treatment (Figure 5C,D). In addition, serum deprivation showed a trend of reducing the CDK7 level, but this difference was not statistically significant and there was no difference in CDK13 level between serum derivation and control. Torin1 alone did not impact CDK7 and CDK13 levels. Regarding the THZ1 treatment, as described in Figure 4C,D above, it similarly reduced the CDK7 and CDK13 level in this experiment (Figure 5C,D). In addition, we found a common trend that serum deprivation or Torin1 in combination with THZ1 had lower levels of CDK7 and CDK13 than THZ1 alone (Figure 5D). The lack of statistical differences between THZ1 and Torin1+THZ1 or serum deprivation+THZ1 was partly due to the variations in the CDK7 and CDK13 protein levels in THZ1 treatment in two independent replicates, given that the combined treatments had a smaller amount of these CDK proteins relative to that in THZ1 treatment in both replicates. For example, compared to THZ1 alone, Torin1+THZ1 in two replicates respectively led to 2.5- and 12.5-fold reduction in CDK7 and 2.3- and 9.1-fold reduction in CDK13. This pattern of further reduction relative to THZ1 was also observed for Ser2P, Ser5P and Ser7P (Figure 5D), although serum deprivation combined with THZ1 led to Ser2P and Ser5P that was barely detectable (Figure 5C). As the total RPB1 level was not affected under this condition, the decrease of Ser2P, Ser5P and Ser7P levels was not likely a result of global protein degradation. With regard to DOCK4 and DOCK9, although we only observed a statistically marginal difference for DOCK4 and a trend for DOCK9 between THZ1 and control in the initial experiment as presented in Figure 4D, THZ1 in this experiment caused a statistically significant 3-6 fold reduction in DOCK4 and DOCK9 levels (Figure 5D). In addition, the combined treatments of THZ1 with Torin1 or with serum deprivation appeared to further reduce DOCK4 and DOCK9 levels compared to THZ1 alone (Figure 5C,D).

## 4. Discussion

### 4.1. Modulation of the Pol II CTD Phosphorylation Code by Rho Family GTPases Is Likely Conserved in Cultured Human Cells via Regulating CTD Kinases and Phosphatases

The shortcut model of Ras or Rho GTPase control of Pol II transcription has been supported so far only by the evidence from the studies of yeast and *Arabidopsis* plant systems [7,8]. In animal cells, there was only one early study involving a rat cardiac cell culture [39], which implicates the impacts of Ras GTPase signaling on the Pol II overall phosphorylation levels, as radioactive isotope labeling (rather than the CTD Ser2P- or Ser5P-specific antibodies) was used. Interestingly, this prior study showed the importance of the CTD domain in Ras activation of transcriptional control and reported a reduction of Cdk7 protein level in a dominant negative Ras mutant and an increase of Cdk7 in a RasGAP (a negative regulator of Ras) mutant, indicating that Cdk7 could be a downstream target of Ras signaling in the control of Pol II transcription. We now provide pharmacological evidence that the CTD Ser2 and Ser5 phosphorylation status could be altered by inhibitors of Ras, as well as Rho, Rac and Cdc42, in human cancer cell lines, supporting a common theme for the Ras superfamily of GTPases-mediated modulation of the Pol II CTD code. Furthermore, our genetic evidence obtained by using siRNA-mediated knockdown of *Rac1* and *Cdc42* convincingly demonstrate a similar modulation of the CTD Ser2 and Ser5 phosphorylation code by these two Rho GTPases. Therefore, our results described here together with the evidence previously reported in yeast and *Arabidopsis* model systems [8] suggest that the shortcut model of Pol II transcriptional control by Rho family GTPases is likely conserved across three of the eukaryotic kingdoms, Animalia, Plantae and Fungi.

Our prior work in yeast and *Arabidopsis* has shown that the proteasome-mediated degradation of a CTD Ser5 phosphatase CPL1 in *Arabidopsis* and a Ser2 phosphatase Fcp1 in yeast is at least part of the mechanism by which Rho family GTPases modulate the CTD Ser2 and Ser5 phosphorylation code [8]. The results reported here suggest differential regulation of CTD phosphatases by Cdc42 and Rac1 in human cancer cell culture. For example, *Cdc42* siRNA knockdown increased accumulation of RPAP2, a CTD Ser5 phosphatase [19], but not FCP1, a Ser2 and Ser5 phosphatase [18], which is an ortholog of yeast Fcp1. This suggests that Cdc42 signaling likely suppresses RPAP2 but not FCP1. In contrast, Rac1 likely suppresses FCP1 but not RPAP2. Note that the Ser specificity for FCP1 and RPAP2 was demonstrated in vitro, but inactivation of the presumably Ser5-specific CTD phosphatase CPL1 in *Arabidopsis* could also lead to an increase of Ser2P level in vivo for the reasons not yet known [8]. Therefore, the decrease of Ser2P and Ser5P levels in *Cdc42* and *Rac1* siRNA-treated cells could be explained at least partially by the increase of RPAP2 and FCP1, respectively. Another possible explanation is the alteration of a CTD Ser5- and Ser7-kinase CDK7 and a Ser2 kinase CDK13, given that both were reduced in *Cdc42* and *Rac1* knockdown cells. Taken together, these results suggest that in cultured human cancer cells, signaling by Cdc42 and Rac1 differentially suppresses CTD phosphatases and commonly increases the level of CTD kinases, which together likely contribute to creation and maintaining of the CTD Ser2 and Ser5 phosphorylation code. One limitation is the use of Ser-specific antibodies in this work, and thus future work using tandem mass spectrometry-based methods [40,41] is needed to profile the dynamic CTD Ser2 and Ser5 phosphorylation status in each peptide repeat along target genes and dissect the mechanisms by which inactivation of Rac1 and Cdc42 controls the level of CTD phosphatases and CTD kinases in an opposite fashion.

### 4.2. A Hypothetical Model for the Rho-Pol II signaling shortcut in transcriptional control in cultured human cells

**Figure 6:**
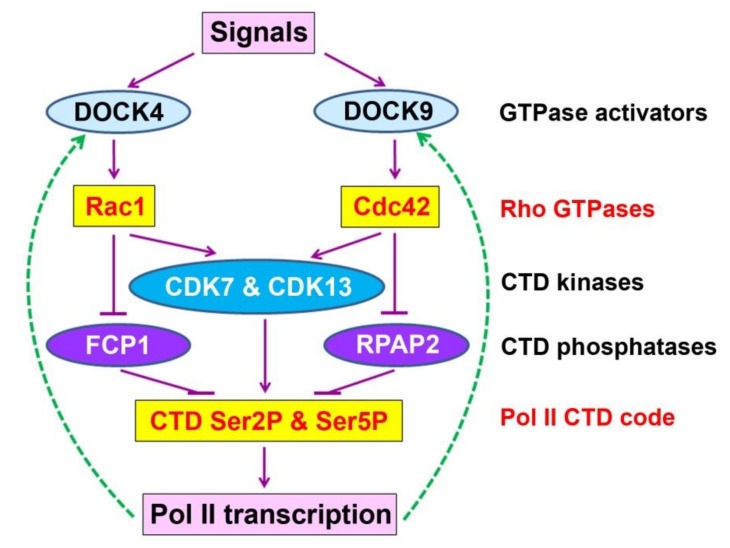
A hypothetical model for the Rho-Pol II signaling shortcut in transcriptional control in cultured human cells. Once activated by extracellular signals, Rac1 and Cdc42 GTPases positively control the degradation of CTD kinases (CDK7 and CDK13) but negatively and differentially control the degradation of CTD phosphatases (FCP1 for Rac1 and RPAP2 for Cdc42) in modulation of the Pol II CTD code (Ser2P and Ser5P). Feedback regulation of DOCK4 and DOCK9, the activators of Rac1 and Cdc42, respectively, is hypothesized to be achieved by transcriptional control of these DOCK genes (indicated by dotted green arrows).

The decrease of DOCK4 and DOCK9 after the THZ1 treatment is an interesting observation in our study. We should point out that the use of two independent biological replicates is a limitation for Western blot experiments in our study and the statistical power would increase if more replicates were performed. Indeed, when two sets of the Western blot results for the THZ1 treatment from Figure 4D and Figure 5D were combined, thus representing a total of four replicates, we found that THZ1 caused a statistically significant decrease in DOCK4 and DOCK9 levels, together with significantly reduced CTD Ser2/4/7 phosphorylation and CDK7/13 accumulation (Supplemental Figure S4). This is different from statistically marginal differences for DOCK4 (Figure 4D) and Ser2P (Figure 5D) or the lack of statistical difference for DOCK9 (Figure 4D). Given the impact of THZ1 in transcriptional control via CDK7 inactivation, the reduction of DOCK4 and DOCK9 is likely caused by transcriptional suppression. DOCK4 has been shown to be a GEF, which activates Rac1 and DOCK9 to be a GEF for activating Cdc42 [27,28,29,30], and thus we hypothesize that the reduction in DOCK4 and DOCK level can potentially lead to inactivation of Rac1 and Cdc42, respectively. Together with the observed suppression of CDK7 and CDK13 by the knockdown of *Rac1* and *Cdc42*, we propose a hypothetical model (Figure 6) in which signaling of Rac1 and Cdc42 to Pol II transcriptional control possibly involves a feedback regulatory loop: Inactivation of Rac1 and Cdc42 leads to inhibition of CDK7 and CDK13 and promotion of FCP1 or RPAP2, which together reduce the CTD Ser phosphorylation status and suppress transcription of *DOCK4* and *DOC9*. Consequently, less DOCK4 and DOCK9 proteins accumulate, which ultimately results in stronger or sustained inactivation of Rac1 and Cdc42. Conversely, activation of these GTPases would be expected to increase CDK7/13, causing up-regulation of DOCK4/9, and as a result stronger activation of Rac1 and Cdc42. This type of feedback regulatory loop would enable the cells to rapidly respond to external stimuli or internal cues in regulating gene expression. Indeed, we have shown that elevation of the CTD Ser2P and Ser5P (caused by the mutation in the *CPL1*-encoed CTD Ser5 phosphatase gene) did lead to activation of ROP GTPase in *Arabidopsis* plants (Supplementary Figure S2), and thus future research is necessary to determine whether reduction of DOCK4 and DOCK9 in human cancer cell culture indeed leads to lower activity of Rac1 and Cdc42, respectively. If this is case, then it will be important to reveal which of Rac1 or Cdc42 signaling components are involved in differential regulation of CTD kinases and phosphatases.

### 4.3. Potential for Developing a Synthetic-Lethal Cancer Therapy

Another intriguing finding from our study is the opposing effects of MG132 and Torin1 on THZ1. THZ1 was discovered as a covalent CDK7 inhibitor [22], with a promising potential in cancer treatment. However, we unexpectedly but consistently observed a reduction of the CDK7 and CDK13 protein level in THZ1-treated cells (Figure 4C,D, Figure 5C,D, and Supplemental Figure S4), which was not reported in several studies that examined CDK7 levels after THZ1 treatment [22,23,24,25,26,42]. In a couple of studies [23,43], although CDK7 protein level was not reduced, a decline in phosphorylated CDK7 was observed instead. In addition, these two studies reported a decrease in the level of other CDKs. For example, one study found a reduction of CDK2 [43]. In the other study, Chipumuro et al. reported a lower amount of both total CDK9 protein and phosphorylated form of CDK9 following the THZ1 treatment, and they interpreted this result as an indirect effect in that THZ1-induced CDK7 inhibition also targets CDK9 transcription [23]. However, one study did report a reduction of CDK7 after THZ1 treatment in one type of peripheral T-cell lymphoma but not in another cell type [44]. In our study, we repeatedly found a 50%-85% reduction of CDK7 and CDK13 levels following 8-hr treatment of THZ1 at a normal concentration (Figure 4C,D and Figure 5C,D). Given that THZ1 might also cross-react with CDK12 and CDK13 at higher concentrations [22], reduction of the CDK13 protein level might not be indirectly caused by CDK7-mediated transcriptional suppression. Importantly, we observed that the THZ1-caused reduction of CDK7 and CDK13 levels could be reversed by the MG132 treatment and enhanced by treatments of Torin1 and serum deprivation, accompanied by the consistent changes in the CTD Ser2P, Ser5P and Ser7P levels and cell number. Therefore, our result strongly implies the involvement of proteasome-mediated degradation of CDK7 and CDK13 by THZ1. Of note, the reversal by MG132 of THZ1 effects was also reported by another study [42]. In that study, Wang et al. found that that MG132 opposed the effect of THZ1 in cyclin B accumulation and chromosomal misalignment, although the CTD Ser phosphorylation status or CDK7 level was not examined. Thus, future work is needed to determine whether THZ1 might interfere with another protein, which is involved in protein degradation and competitively bound by MG132, or whether THZ1 indirectly causes transcriptional upregulation of a proteasome component, which is subjected to MG132 blocking.

Most importantly, we found that combining THZ1 with Torin1 or serum deprivation had a trend of further reducing CDK7/13 and Ser2P/5P/7P levels and caused the death of much larger number of cultured cancer cells compared to THZ1 alone. Although Torin1 by itself (up to 100 nM) had no impact in our treatment regime, it could significantly enhance the THZ1 effect, even at much lower doses (5-10 nM). Such synergistic effects of THZ1 with other compounds that alter p53 transcription, receptor tyrosine kinase or hedgehog pathways were recently reported [45,46,47]. It is possible that the proposed Rac1 and Cdc42 feedback regulatory loop is involved, as their activators DOCK4/9 are also further reduced by the combined treatment of Torin1 and THZ1. Therefore, our finding might form an important basis for developing a potential synthetic lethal therapeutic strategy that targets the activity or degradation of CDK7 and other protein components involved in the Rho GTPase signaling shortcut model proposed here.

## 5. Conclusions

In plant and yeast systems, Rho GTPase signaling has been shown to link to Pol II transcription by modulating the CTD code, but whether this shortcut model is conserved in humans remains completely unknown. We provided pharmacological and genetic evidence showing a similar Pol II CTD code modulation by Cdc42 and Rac1 GTPases in cultured human cancer cells. Thus our work reported here suggests a likely universal signaling shortcut from Rho to Pol II across the three eukaryotic kingdoms, Fungi, Plantae and Animalia, via regulation of distinct CTD phosphatases but common CTD kinases. Additionally, combining the treatments that promote protein degradation and a CDK7 inhibitor THZ1 has the potential for developing a synthetic-lethal therapy for more effective cancer treatment.

## Supplementary Materials

The following are available online at https://www.mdpi.com/2073-4409/9/3/621/s1, Figure S1. Quantitative analysis of cell number after Rac1 and Cdc42 siRNA treatments. Figure S2. Activation of ROP GTPase in the loss-of-function cae2-1 allele of the CTD Ser5 phosphatase-coding CPL1 gene in Arabidopsis plants. Figure S3. A dose response of Torin1 in enhancing the THZ1 effect of decreasing cell number. Figure S4. The impact of THZ1 on CTD Ser2/5/7 phosphorylation, CDK7/13 and DOCK4/9 levels analyzed by the aggregated data.
