# Supplementary material for: Modulation of the Pol II CTD Phosphorylation Code by Rac1 and Cdc42 Small GTPases in Cultured Human Cancer Cells and Its Implication for Developing a Synthetic-Lethal Cancer Therapy
Source: Cells. 2020 Mar 4;9(3):621. doi: 10.3390/cells9030621 (PMC7140432; doi:10.3390/cells9030621)
Supplement: Supplementary file 1 [file cells-09-00621-s001.zip › Figure S1_v2.pdf]

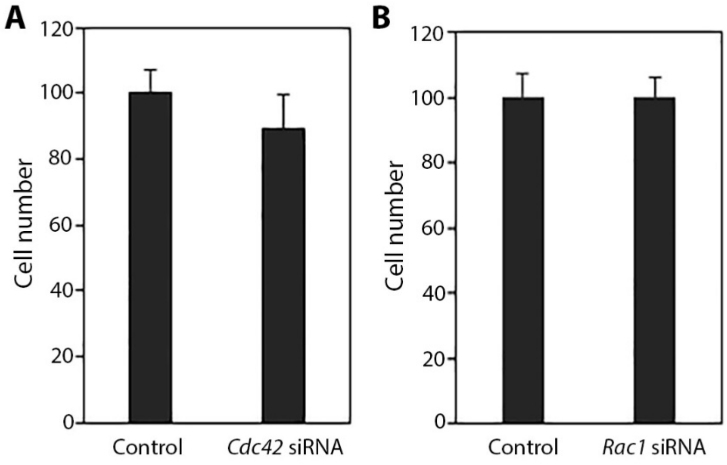

Figure S1. Quantitative analysis of cell number after *Rac1* and *Cdc42* siRNA treatments. (*A*) *Cdc42* siRNA. (*B*) *Rac1* siRNA. Cell number for the control average is set as 100, and all other treatment groups are normalized to the control. Values are means and SEM of five technical replicates from one representative experiment.
