# Supplementary material for: Modulation of the Pol II CTD Phosphorylation Code by Rac1 and Cdc42 Small GTPases in Cultured Human Cancer Cells and Its Implication for Developing a Synthetic-Lethal Cancer Therapy
Source: Cells. 2020 Mar 4;9(3):621. doi: 10.3390/cells9030621 (PMC7140432; doi:10.3390/cells9030621)
Supplement: Supplementary file 1 [file cells-09-00621-s001.zip › Figure S2.pdf]

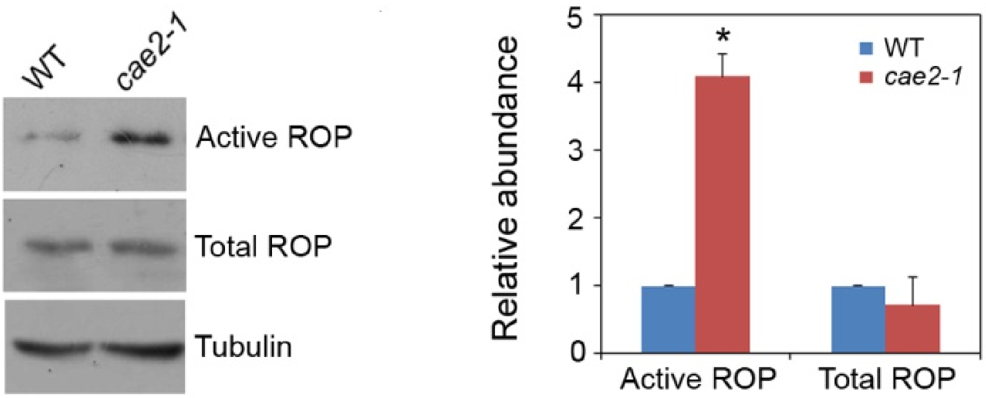

Figure S2. Activation of ROP GTPase in the loss-of-function *cae2-1* allele of the CTD Ser5 phosphatase-coding *CPL1* gene in Arabidopsis plants.

Protein extracts were prepared from young seedlings of Arabidopsis wild-type (WT) and the *cae2-1* allele of the *CPL1* gene. The GTP-bound, active from of ROP proteins were pull down by use of MBP-RIC1 resins and detected using Western blot with a ROP antibody (Sigma; R9529) which recognizes ROP2 and other members of ROP GTPases, by following the protocol described elsewhere (Xu et al., 2010. *Cell* 143: 99–110). Prior to pull-down, a small fraction of protein extracts was used in Western blot to detect the level of total (i.e. both GTP- and GDP-bound forms) ROP proteins. Tubulin, an internal loading control. Left panel shows Western blot images from one replicate, and the quantitative analysis result of protein abundance from two independent replicates is presented in the right panel. Protein level in the control was set as 1.0. Significance level is indicated by * (p<0.05) vs. WT. As our prior work has shown that activation of ROP2 GTPase promotes CPL1 protein degradation, leading to elevated Pol II CTD Ser2P and Ser5P level (Zhang et al., 2016. *PNAS* 113, E8197-E8206), this observation that loss of CPL1 function causes a 4-fold increase in the level of active form of ROP GTPase (without affecting total ROP protein level) suggests that ROP2 GTPase signaling likely exerts a positive feedback regulatory loop in the ROP2 GTPase-mediated modulation of the Pol II CTD Ser phosphorylation code.
