# Supplementary material for: Modulation of the Pol II CTD Phosphorylation Code by Rac1 and Cdc42 Small GTPases in Cultured Human Cancer Cells and Its Implication for Developing a Synthetic-Lethal Cancer Therapy
Source: Cells. 2020 Mar 4;9(3):621. doi: 10.3390/cells9030621 (PMC7140432; doi:10.3390/cells9030621)
Supplement: Supplementary file 1 [file cells-09-00621-s001.zip › Figure S3.pdf]

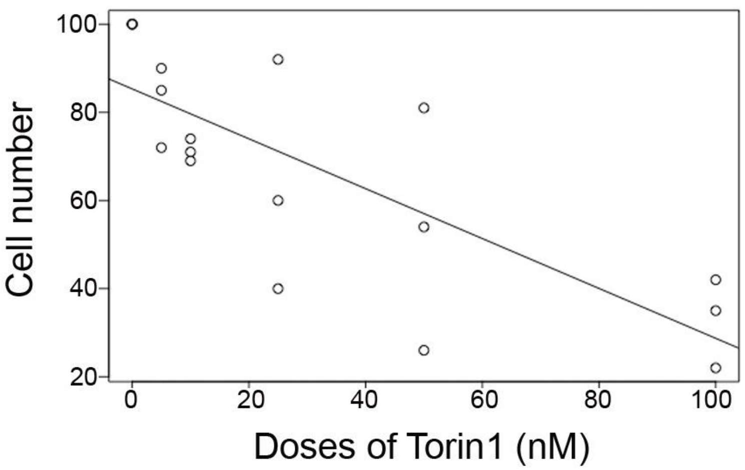

Figure S3. A dose response of Torin1 in enhancing the THZ1 effect of decreasing cell number. Cells were treated with 0, 5, 10, 15, 50 and 100 nM of Torin1 in combination with 100 nM THZ1 for 48 hr. Shown are the means of relative cell number for each of the three replicates (each replicate having three measurements), with the 0 nM Torin1 control being set as 100%. Linear regression analysis was performed.
