# Supplementary material for: Modulation of the Pol II CTD Phosphorylation Code by Rac1 and Cdc42 Small GTPases in Cultured Human Cancer Cells and Its Implication for Developing a Synthetic-Lethal Cancer Therapy
Source: Cells. 2020 Mar 4;9(3):621. doi: 10.3390/cells9030621 (PMC7140432; doi:10.3390/cells9030621)
Supplement: Supplementary file 1 [file cells-09-00621-s001.zip › Figure S4_v2.pdf]

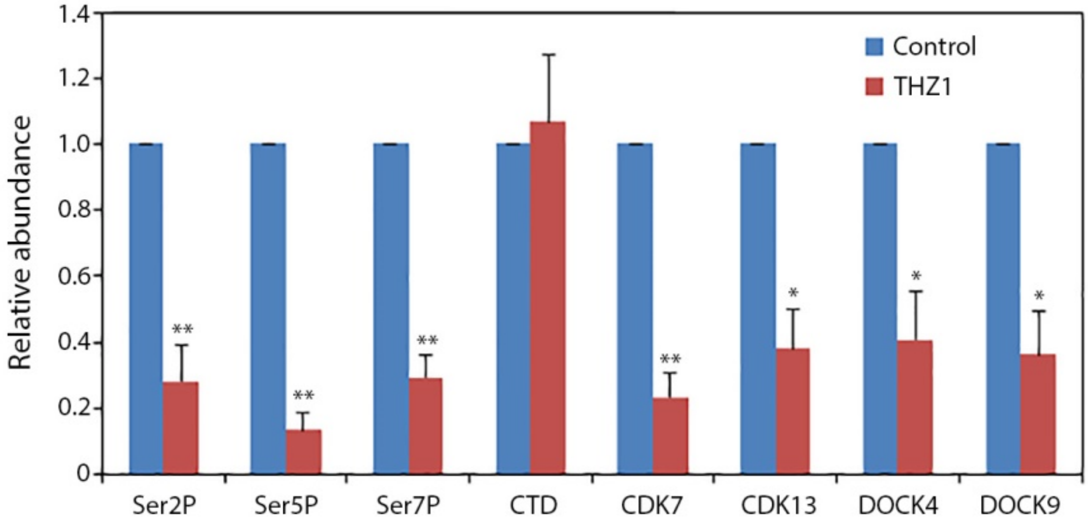

Figure S4. The impact of THZ1 on CTD Ser2/5/7 phosphorylation, CDK7/13 and DOCK4/9 levels analyzed by the aggregated data.

The Western blot results on the THZ1 treatment, which were obtained by combining the data in Figures 4D and 5D, representing two biological replicates from each of two independent experiments, with a combined total of four replicates, were re-analyzed with statistical test. Protein level in the control for each replicate was set as 1.0. Significance level is indicated by * (p<0.05) or ** (p<0.01) vs. control.
